# End-of-Life Care Preferences of Patients with Advanced Urological Malignancies: An Explorative Survey Study at a Tertiary Referral Center

**DOI:** 10.3390/curroncol31010031

**Published:** 2024-01-12

**Authors:** Christian Volberg, Fabian Urhahn, Anna J. Pedrosa Carrasco, Astrid Morin, Martin Gschnell, Johannes Huber, Luka Flegar, Hendrik Heers

**Affiliations:** 1 Department of Anesthesiology and Intensive Care Medicine, University Hospital Marburg, Philipps University Marburg, Baldingerstraße, 35033 Marburg, Germany; 2 Research Group Medical Ethics, Faculty of Medicine, Philipps University Marburg, Baldingerstraße, 35033 Marburg, Germany; 3 Department of Neurology, Philipps University Marburg, Baldingerstraß, 35033 Marburg, Germany; 4 Department of Dermatology and Allergology, Skin Tumor Center, University Hospital Marburg, Philipps University Marburg, Baldingerstraß, 35033 Marburg, Germany; 5 Department of Urology, University Hospital Marburg, Philipps University Marburg, Baldingerstraß, 35033 Marburg, Germany

**Keywords:** urological malignancies, end of life, palliative care, patient preference, place of death

## Abstract

**Background:** Many people want to die at home, but it is often not possible because they do not share their wishes with family members. This study was conducted to find out the extent to which patients with advanced urological malignancies had wishes regarding their final stage of life, made arrangements accordingly, and communicated their wishes to relatives and health care professionals. **Methods:** We conducted a survey among advanced urological tumor patients during their clinic visit at a German university hospital using a 31-item questionnaire. Inclusion criteria were metastatic or irresectable prostate cancer, urothelial carcinoma, or renal cell carcinoma. **Results:** In total, 88 patients (76 male, 12 female) completed the questionnaire, and 62 of those respondents (70%) had received their tumor diagnosis within the past 5 years. Symptoms were reported by 80%, and 18% described five or more symptoms. The majority (88%) stated that they had thought about their preferred place of death but 58% had not informed anyone about it. The preference for a hospice as the place of death correlated statistically significantly with the absence of a domestic partnership (*p* = 0.001) or marriage (*p* < 0.001) and with a high number of symptoms (≥5; *p* = 0.009). However, 73% had not talked with their urological oncologist about care options in case their health deteriorated though 36% of those were interested in having a conversation about it. **Conclusions:** Our results showed that 9 out of 10 patients reflected on their preferred place of death but only a few discussed it with anyone. Based on this finding, physicians and healthcare staff should initiate discussions about early care planning so that patients in incurable situations can express their wishes regarding their preferred place of death.

## 1. Introduction

Modern tumor therapy helps patients in advanced stages of urological malignancy to survive for increasingly longer periods of time [1]. Nevertheless, many patients experience distressing symptoms of their malignancy until they die. In this final stage of life, between 58% and 93.8% of the overall population in Germany wishes to remain in their home environment and die there [2,3,4,5]. An important goal of modern palliative medicine in the care of terminally ill people is to fulfill this wish. However, previous findings have suggested that, in reality, only 21% of patients actually pass away at home and that the number of deaths at home has decreased over the past 20 years [6].

A previous study showed that, especially in cancer patients, quality of life could be improved in the last phase of life by co-caring palliative physicians if wishes regarding the last phase of life were discussed with patients at an early stage [7]. However, such discussions often do not take place. Patients are often over-treated, and the patient’s wishes regarding their place of death are not discussed [8]. Several barriers that affect communication regarding the end of life can hinder patients and their families as well as treating physicians from discussing their wishes regarding the last phase of life. Moreover, physicians often feel unprepared to communicate with their patients about such difficult topics [9].

In palliative care, having choices and control over one’s place of death is seen as essential for a “good death” [10]. End-of-life planning can reduce stress in the person concerned, such as by eliminating unresolved problems. It can also make things easier for family members, who do not have to make decisions that may be overwhelming. A living will and advance care planning (ACP) can be helpful tools in this context [9].

Data on the preferred place of death have been provided by several studies in Germany [11,12,13]. In these studies, more than half of the respondents indicated that they preferred to die at home. The factors that influence the decision on the favored place of death are diverse and heterogeneous [14]. In Germany, various studies have shown that the majority of deaths take place in medical facilities [2,3,4,5]. Current data on hospital deaths are collected by the Federal Statistical Office (DESTATIS). According to these data, 427,199 people died in hospitals in 2019, which was 45.5% of a total of 939,520 deaths in Germany [15,16]. The majority of patients with a malignant tumor disease are among those to die in hospital, but the rate of deaths in a hospice or at home is higher than in the general population [17].

This study aimed to determine the extent to which patients with advanced urological malignancies had preferences for their final phase of life, whether they had planned accordingly, and whether they had communicated their wishes to relatives and medical professionals.

## 2. Methods

This study was conducted with patients treated at the oncology clinics of the urology department at a German tertiary referral center from March to December 2021. Patients with metastatic or irresectable prostate cancer, urothelial carcinoma, or renal cell carcinoma were asked to participate in the survey while waiting for consultation. A research assistant was available to answer any questions or provide assistance. The number of patients who did not agree to participate in this study was recorded for statistical evaluation. After providing informed consent to participate in this study, the patients completed a 31-item questionnaire covering several sub-areas:

- Demographic data;
- Preferences regarding the preferred place of death;
- Existence of a living will and/or health care proxy;
- Level of knowledge about their disease;
- Communication about wishes for the last stage of life.

The questionnaire was prepared through an iterative development process by the authors based on a similar questionnaire previously used by our group in a skin cancer cohort [18]. During this process, the wording and order of the questions were optimized. Finally, 15 volunteers tested the questionnaire, which showed good face validity. 

The present study was approved by the local ethics committee of Philipps University Marburg (file number: 34/21) and registered in the German Clinical Trials Register (DRKS Reg. No.: 00025957). Data analyses were conducted using SPSS^®^ Statistics, version 27 (IBM corp., Armonk, NY, USA). Several qualitative characteristics were arranged in contingency tables and assessed for dependence with chi^2^ tests and, in case of dichotomous manifestations, with Fisher’s exact test. The significance level was specified as alpha ≤ 0.05. 

## 3. Results

### 3.1. Demographics and Symptoms

Ninety-eight patients were identified who met the inclusion criteria. Of these, 88 patients (90%) were recruited for participation (4× lack of ability to consent, 2× insufficient knowledge of German, 4× refusal by patients). The participants were predominantly male (76; 86%); age range 47–87 years, mean age 70.2 years (SD 9.05 years). Further demographic data as well as the gender-dependent distribution of tumor entities are provided in Table 1.

Thirteen respondents (15%) had a second malignancy in addition to the urological tumor disease. The majority of the participants had received their tumor diagnosis within the past 5 years. 

Symptoms were reported by 70 (80%) respondents while 18 (21%) had no symptoms at the time of the survey. Of the symptomatic respondents, 12 were women (100% of female respondents) and 58 (76%) were men.

Participants could report multiple symptoms: 82% indicated up to four symptoms, and 18% described five or more symptoms (see Table 2). The most frequent symptoms were pain (61%), sleep disturbance (50%), diarrhea/constipation (43%), and sexual problems/reduced libido (31%). The latter symptom was particularly prevalent among the male respondents (96%). Beyond those, 31% reported other symptoms. Of the 70 respondents who described symptoms, 51 (73%) reported being burdened by these symptoms. That sense of burden was more prevalent among women (75%) while only 55% of men reported the same feeling.

### 3.2. Preferred Place of Death

Of the 88 respondents, 77 (88%) had a preferred place of death while 11 respondents (12%) had no preference. At 73% (64 respondents), “at home” was by far the most common place of death preference. Thirteen respondents (15%) had a preferred place of death other than “at home”, of which “hospice” was clearly the most common response at 10%. Interestingly, no patients indicated that they wanted to die in a palliative care unit. Notably, men wanted to die at home more often than women did, while women preferred to die in hospital or a hospice more often than men did. The frequency distributions of the desired place of death by the number of symptoms and civil partnership are shown in Table 3. 

There were statistically significant associations between having a preferred place of death and being female (*p* = 0.015), a high symptom burden of five or more described symptoms (*p* = 0.027), being married (*p* = 0.002), or being in a domestic partnership (*p* < 0.001). Individuals with a higher educational level, grouped into (technical) university degree, completed vocational training, and no professional training, showed a higher likelihood of having a preferred place of death (*p* = 0.021).

The desired place of death “at home” (*n* = 64) showed statistically significant correlations with being in a civil partnership (*p* = 0.006) or married (*p* = 0.015), and with a low number of symptoms (< 5; *p* = 0.033).

The preference for a “hospice” (n = 9) correlated statistically significantly with the absence of a domestic partnership (*p* = 0.001) or marriage (*p* < 0.001), and with a high number of symptoms (≥ 5; *p* = 0.009), as well as with the presence of advanced care documents (*p* = 0.013).

### 3.3. Communication about Place of Death

Fifty-one respondents (58%) had not informed anyone about their preference. Thirty-five (40%) had talked to a relative/friend, family doctor, or both. Two respondents (2%) did not remember whether they had told anyone.

The majority of respondents who thought about dying in a hospice (9 respondents) had communicated this (56%). Among respondents who wished to die at home (*n* = 64), a minority (42%) had communicated their desired place of death.

Twelve patients (46%) with a long-standing diagnosis (> 5 years) had communicated their preference, as compared with 23 patients (37%) with a diagnosis in < 5 years, which was not a statistically significant difference. 

### 3.4. Living Will/Health Care Proxy

Fifty-six respondents (64%) had a health care proxy and fifty-one (58%) had a living will. Forty-six respondents (52%) had both documents. Among the respondents, 30% (*n* = 26) knew that they had not generated either document, and one respondent did not remember. 

However, the proportion of respondents who had prepared at least one of the precautionary documents was higher among those whose tumor diagnosis was more than 5 years ago than among those who had been diagnosed with a tumor more recently (77% vs. 66%). Only 13 (37%) of the 35 respondents who had communicated their desired place of death had documented it in their living will.

**Figure 1:**
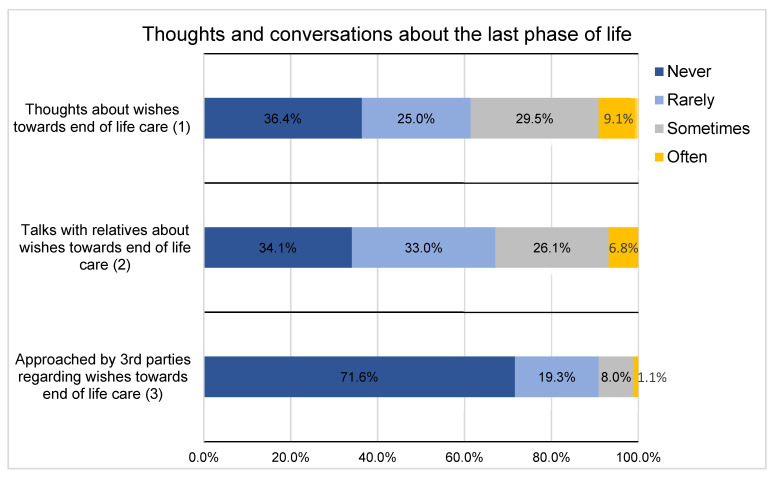
Preoccupation with wishes for the last phase of life.

A similar number of respondents stated that they thought about their wishes for the last stage of life at least occasionally (a total of 56 respondents (64%)) or talked about it with relatives (a total of 58 respondents (66%)). Among the respondents, 29.5% (*n* = 26) said they had been approached by at least one person with regard to this topic. Meanwhile, more than two-thirds (*n* = 62; 71%) indicated that they had not yet been asked about their wishes for the last phase of life. When patients were approached about the topic, this was most frequently by family members: 62% (*n* = 16) by their partner and 39% (*n* = 10) by their children. Multiple answers were possible, so that 10 respondents (39%) stated that they had talked with more than one group of people about their wishes for the last phase of life (Figure 1).

### 3.5. Discussions about Care Options

Only 24 respondents (27%) had talked to their urological oncologist about care options in case their health deteriorated. Among the rest, 36% were interested in having an informative discussion about care and treatment options in the event of worsening health, whereas 50% said they would not want such a talk, and 14% were undecided.

## 4. Discussion

Most advanced stage urological cancer patients have a preferred place of death. However, the majority never discloses their wish to relatives or their medical care team. While half of the patients make arrangements for a living will and/or health care proxy, there still is a considerable percentage who do not discuss the matter of dying and advanced care planning with anyone. 

Systemic treatment options for patients with advanced tumor diseases affect their survival and quality of life. Nonetheless, these patients will eventually die from tumor disease. In preparation for the end of life, according to individual wishes, national and international data show that the majority of people want to die at home [4,12,14,19,20,21].

Gomes et al. showed a preference for dying at home among 31–87% of cancer patients [19]. Our study demonstrated similar results: 73% of patients with advanced urological malignancy said that they wanted to die at home. It is interesting to note that men and women have different preferences for their place of death. Men are more likely to want to die at home while women are more likely to want to die in a hospital or hospice. A possible explanation for this finding could be that there is a fundamental difference in life expectancy between men and women, and that women live longer than men. Men are more likely to die of serious illnesses while women live longer with chronic illnesses and the potential need for care [22,23]. Furthermore, in traditional gender roles, men may expect to receive care at home through their family, especially their wife, whereas women are more aware of a potential need for professional care assistance and do not expect to receive care through their husband/life partner. Their awareness of the potential for their dependency on care in the future might be reflected in the fact that compared with men, women are more likely to consider hospice or hospital care at the end of life.

In theory, access to specialized palliative care at home and instruments of care, such as wheel chairs, hospital beds, and shower modifications, could also be worrisome for patients when they become increasingly symptomatic and dependent on others. In Germany, we are fortunate to have excellent infrastructure and a legal right to palliative care and easy access to equipment. In our region, specialized ambulatory palliative care (German abbreviation: SAPV) is broadly available and offered by teams of caregivers and physicians with expertise in palliative care. This network helps to reduce the need for hospital treatment in palliative patients by offering treatment and support at home [24]. 

Only when cancer patients have thought about the final phase of life and communicated their thoughts can relatives and medical care providers work toward fulfilling these wishes. Just under half of the respondents who had formulated a preferred place of death had communicated this wish to a third party. The proportion of respondents who had communicated their preference to someone did not increase significantly as the time interval from the tumor diagnosis increased. The results showed that patients prefer to die at home as long as they are not seriously ill or dependent on help from others. Those with a high symptom burden (≥ 5 symptoms) are more likely to prefer to die in a hospice and share this wish with their family. The same trend was observed among patients who live alone since most people do not want to die alone; instead, they want other people around them when their time comes or when they recognize, especially when suffering from a high symptom burden, that they need care support or felt safer with the medical care a hospice provides. Similar results were observed in patients with advanced skin cancers. In these patients, a higher symptom burden or advanced stage of illness provides a reason for wanting to die in a hospice [18]. In the literature, comparable results were shown for people who are chronically ill or living alone, and Fereidouni described that a change in the preferred place of death is dependent on demographic, disease-related, and psycho-social variables [14,25]. 

The purpose of writing a living will or health care proxy is to ensure that the wishes and ideas of the patient take effect in the event of their incapacity to give consent [26]. Here, 69% of the respondents had drawn up at least one of these documents, and 52% had prepared both. The prevalence of precautionary documents in our cohort was higher than in previous representative German population surveys [11]. We also found that a higher proportion of respondents whose tumor diagnosis was made > 5 years ago had created at least one document compared to respondents with a more recent diagnosis. This is a positive development, as it shows that patients are coming to terms with their illness and making provisions for the last phase of their lives. In order to avoid overtreatment in the final stage and to implement the patient’s individual wishes, a living will is helpful [27,28]. Another instrument is advance care planning (ACP), in which an individualized living will is designed in detailed discussions with specially trained staff. One advantage of ACP is that relatives are encouraged to be present during its preparation and hear the wishes of the patient, which helps to avoid misunderstandings and misconceptions in the family [9,29]. Patients should be subtly offered the opportunity to complete a living will, health care proxy, or ACP. Posters or brochures about the necessity of these documents in the waiting area are suitable for this purpose, but the treating physician should also actively ask about the existence of these documents and offer to help patients complete them.

Brighton and Bristowe [30] described the importance of end-of-life care conversations and the benefits patients can derive from early care planning. In our study, 64% of respondents reported thinking at least infrequently about their wishes for the last phase of life, and 66% had talked with family members about these wishes. However, only 28% had been asked by someone outside the family about their wishes for the last phase of life. Three respondents indicated that this approach had been made by the family physician. Only 25% had been approached by their urological oncologist about treatment and care options in the event of deteriorating health. One-third of the other respondents expressed an interest in such a discussion. These findings indicate that there is a need among a significant proportion of patients to discuss treatment and care options, as shown in other studies of advanced-stage cancer patients [31]. Although 50% of the respondents were not (yet) willing to have such discussions, the treating physician should determine in the patient interview whether there is a need for ACP on the part of the patient. Discussions about the end of life should be initiated by the urological oncologist, as studies have shown that the early involvement of palliative care staff leads to an improvement in quality of life and longer survival [27,32]. However, in both patients and their physicians, barriers impair communication, so it could be helpful if such discussions were an integral part of the treatment process [9]. For medical staff, the ”surprise question” (“Would I be surprised if this patient were to die in the next 12 months?”) is a suitable marker to initiate end-of-life conversations if the answer, in self-reflection, is “No, I would not be surprised” [33]. Nevertheless, it is also important to note that simply recognizing the problem is not enough; physicians also need to be trained in communicating about end-of-life discussions and wishes for the last phase of life. The relatively new concept of the *Serious Illness Conversation Guide* should be mentioned here, which can be a helpful tool in discussing end of life care [34,35]. Conversations about the final phase of life should not be offered only once to patients. It should be understood by patients and relatives that potential issues and changes in the patient’s wishes can always be addressed during all stages of the treatment process. The initial offer of such a conversation should be seen as a means of lowering the barriers to discussing this difficult topic. 

It is important for physicians and nurses involved in cancer care to acknowledge the discrepancies between patients’ wishes about their end of life and the treatment reality. Nonetheless, care teams should encourage patients to talk about their preferences and take those wishes into account. The involvement of specialized palliative care teams can be beneficial in that regard. 

## 5. Limitations and Strengths

This study is limited by its single-center design and the relatively small sample size. Furthermore, the questionnaire used was not formally validated. The findings cannot be generalized to the rest of Germany or other countries because some aspects of communication between patients and their families and health care providers may be influenced by cultural factors and protocols specific to a health care system or workplace. In future surveys, it would be interesting to determine the proportion of patients who want to die at home and are able to do so. For this purpose, it would be necessary to determine whether spatial conditions and supporting relatives are available so that this wish can be fulfilled. Unfortunately, our questionnaire did not include these factors.

Nevertheless, there is very little research on the final stage of life in urological cancer patients, and the findings of our questionnaire specifically designed for this topic provide important novel insights.

## 6. Conclusions

Our analysis showed that 9 out of 10 patients with incurable urological cancer had thought about their preferred place of death, but only a few had communicated their wishes to another person. Therefore, greater emphasis should be placed on early care planning discussions by treating physicians and nurses so that patients are encouraged to express their end-of-life wishes. Furthermore, treating physicians and nurses should be trained in communicating end-of-life discussions. Finally, as people living alone or with a high symptom burden are more likely to want hospice care, this should be offered as an option.

## Figures and Tables

**Table 1:** Demographics.

		*n*	%
Gender	Male	76	
	Prostate cancer	41	54
	Renal cell carcinoma	26	34
	Urothelial carcinoma	9	12
	Female	12	
	Renal cell carcinoma	9	75
	Urothelial carcinoma	3	25
Age	<70	42	48
	≥70	46	52
Time since tumor diagnosis	<5 years	62	70
	Prostate cancer	29	33
	Renal cell carcinoma	21	24
	Urothelial carcinoma	12	14
	≥5 years	26	30
	Prostate cancer	12	14
	Renal cell carcinoma	14	16
	Urothelial carcinoma	0	0
Tumor entity	Prostate cancer	41	47
	Renal cell carcinoma	35	40
	Urothelial carcinoma	12	14
Symptoms	Symptoms	70	80
	No symptoms	18	20
Number of symptoms	<5	72	82
	≥5	16	18
Symptom burden	Affected	51	58
	Not affected	37	42
Marital status	In a stable relationship or married/registered partnership	66	75
	Single/widowed	22	25
Need for care	Care requirements	33	38
	No need for care	55	63
Education	(Technical) university degree	12	14
	Vocational training	69	78
	No professional training	7	8

**Table 2:** Symptoms according to tumor entity.

	Tumor Entity				
Symptoms	Prostate Cancer	Renal Cell Carcinoma	Urothelial Carcinoma	Total	% of Cases
Pain	21	17	5	43	61.4
Shortness of breath	2	10	3	15	21.4
Urinary tract infection	2	1	2	5	7.1
Visible hematuria	1	0	2	3	4.3
Sexual problems/ reduced libido	15	5	2	22	31.4
Nausea	4	11	2	17	24.3
Diarrhea/constipation	10	14	6	30	42.9
Sleep disorders	15	13	7	35	50.0
Pronounced daytime tiredness (fatigue)	4	8	2	14	20.0
Open wounds	1	5	0	6	8.6
Fear	6	4	4	14	20.0
Other symptoms	8	12	2	22	31.4

**Table 3:** Preferred place of death depending on civil partnership and number of symptoms.

		Civil Partnership						Number of Symptoms			
		Single, Widowed	% of Life Partnership	% of Death Place Request	Stable Relationship, Married/Registered Civil Partnership	% of Life Partnership	% of Death Place Request	<5 Symptoms	% from < 5 Symptoms	≥5 Symptoms	% from ≥ 5 Symptoms
Preferred place of death	At home	11	50.0	17.2	53	80.3	82.8	56	77.8	8	50.0
	Nursing home	1	4.5	100.0	0	0.0	9.1	1	1.4	0	0.0
	Hospital	0	0.0	0.0	2	3.0	100.0	1	1.4	1	6.3
	Hospice	7	31.8	77.8	2	3.0	22.2	4	5.6	5	31.3
	Other place	1	4.5	100.0	0	0.0	0.0	1	1.4	0	0.0
	Not important	2	9.1	18.2	9	13.6	81.8	9	12.5	2	12.5
	Total	22	100	25	66	100	75	72	100	16	100

## Data Availability

The data presented in this study is available on request from the corresponding author.
